# Color Reversion of Refined Vegetable Oils: A Review

**DOI:** 10.3390/molecules28135177

**Published:** 2023-07-03

**Authors:** Xiaozhong Chen, Shangde Sun

**Affiliations:** College of Food Science and Technology, Henan University of Technology, Zhengzhou 450001, China; xiaozhongchen521@163.com

**Keywords:** color reversion, oxidation, refined vegetable oil, γ-tocopherol, tocored

## Abstract

During the transport, storage, and consumption of edible vegetable oils, the color of some freshly refined oils is gradually darkened, which is known as the color reversion. The oil industry has been plagued by the issue for a long time because the dark color of the oil is related to its poor quality and low acceptability for consumers. Color reversion of refined vegetable oils is primarily related to the processing pigments, especially tocored, which is the oxidation product of γ-tocopherol. However, the underlying molecular action mechanism of tocored is not yet fully understood due to the complex transformations of tocored in oil systems. This paper presents a brief description of oil color, followed by an overview of research progress on the mechanism of color reversion. In particular, the effect of minor components (phospholipids and metal ions) on color reversion is highlighted in an attempt to explain the remaining mysteries of color reversion. Furthermore, the measures to restrain color reversion by quality control of the oilseeds, the adjustment of technical parameters of processing, and the storage conditions of refined oils are summarized to provide some references for the oil industry.

## 1. Introduction

Refined vegetable oils, after degumming, deacidification, bleaching, deodorization, and dewaxing, appear pale yellow or amber when they are freshly prepared. However, during transport, storage, and consumption, the color of some vegetable oils gradually darkens and turns deep yellow or light red. This phenomenon is known as the color reversion of oils [1]. The color of oils is typically described as red and yellow values by Lovibond Tintometers in the edible oil industry, and the degree of color reversion of oils is referred to by the variation of red values [2].

The gradually deepening color of freshly prepared oils was due to oxidation. Meanwhile, the peroxide value of the oil, an indicator of the content of primary oxidation products, was significantly increased during the deepening process, which resulted in the poor quality of the oil [1]. A similar deepening color phenomenon is found in frying oil during deep-fat frying [3]. Furthermore, refined vegetable oils in light colors are more favored by consumers than those in dark colors, as light colors are considered to be associated with freshness. Therefore, the dark color of refined vegetable oils resulting from color reversion is related to poor quality and low acceptability for consumers [4,5]. Besides these, refined vegetable oils are commonly treated as cooking oils by Asian consumers, and their color changes are pronounced due to the traditional transparent packaging [6]. As a result, it is important to maintain the stable appearance of refined vegetable oils, especially to restrain the color reversion of oils.

The studies on the color reversion of refined vegetable oils can be divided into four stages [1]. The first stage was from 1922 to 1960. During this stage, Sievers and McIntyre [7] and Dechary et al. [8] found that the color reversion of oils was correlated with free fatty acids (FFA) and gossypols, respectively. However, Swift et al. [9] and Nakamura et al. [10] found that the oxidation product of γ-tocopherol, γ-tocopherol-5,6-quinone (tocored), was responsible for the color reversion of oils.

The second stage was from 1960 to 1980, and the research was dominated by Japanese researchers. Tomita et al. [11] found that the degree of color reversion of the refined soybean oil was positively correlated with the moisture content of raw soybean seeds. The relationship between raw soybean moisture, tocored, and color reversion has been studied by Komoda et al. [12,13]. The authors reported that the degree of color reversion was determined by the amount of tocored, which in turn was influenced by the moisture content of the raw soybean seeds. Furthermore, a mechanism of color reversion was proposed according to the variation of the tocored content during the refining of soybean oil.

The third stage was from 1980 to 2000, and the research was concentrated in Taiwan, China. A color reversion mechanism, which was significantly different from that of Japanese researchers, was proposed by Lai et al. [14]. Subsequently, Chu and Lin [15] introduced a method for the pretreatment of raw soybeans to improve the color stability of soybean oil.

The fourth stage was from 2000 to now. The correlative works were mostly concentrated in China, and the research field was divided into two parts: mechanism analysis and restraining measures. The color reversion of vegetable oils has been systematically studied by Dr. Zhang [16]. The author reported that the color reversion of oils might be attributed to the interaction of tocored and oxidized triacylglycerols (OX-TAG). In addition to the mechanism, measures to restrain color reversion have also been proposed. The measures were focused on the following five aspects:

(I) The quality control of oilseeds. For example, the degree of color reversion of oils was influenced by the cultivar, the moisture content, and the maturity of the oilseeds.

(II) The pretreatment of oilseeds. Steam pretreatment of raw soybeans has been proven to be an effective measure to restrain the color reversion of refined soybean oil [15].

(III) The oil extraction process. The temperature during the process was very important for the color stability of the oil.

(IV) The refining of crude oil. The five processes involved in vegetable oil refining are degumming, deacidification, bleaching, deodorization, and dewaxing. The color reversion of refined vegetable oils was influenced by the technical parameters of these processes (except dewaxing).

(V) The storage of refined vegetable oils. The speed of color reversion of refined vegetable oils was determined by the addition of exogenous antioxidants as well as the temperature, light, and sealing conditions during storage.

In this review, the color of vegetable oils and the research progress on the mechanism of color reversion were presented. To understand the reasons for color reversion, the effect of minor components was highlighted. In addition, the restraining measures were also summarized to provide some available references for subsequent research and the oil industry.

## 2. The Color of Vegetable Oils

The color of vegetable oils is mainly attributed to two types of pigment. One is the naturally existing lipid-soluble pigments, such as carotenoids and chlorophylls. The other is the pigments produced during oil processing, which are called the processing pigments, for example, the products of oxidative polymerization, and non-enzymatic browning reactions.

### 2.1. Natural Pigments

**Figure 1:**
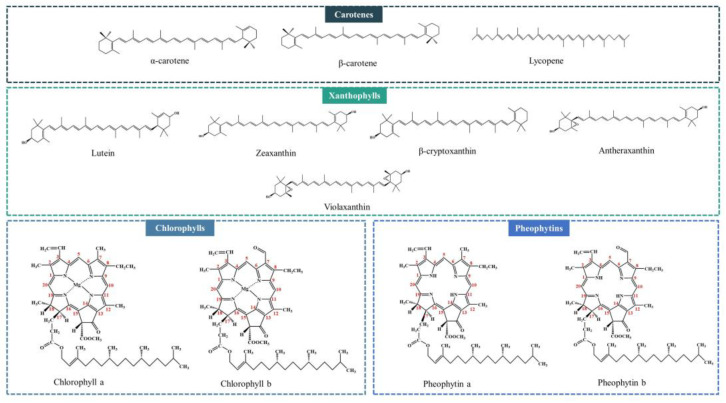
Structures of major natural pigments in oils.

Carotenoids and chlorophylls are the predominant natural pigments present in most vegetable oils [17]. Carotenoids are typically composed of a C40 skeleton (tetraterpenoids), which are responsible for the red, yellow, and orange colors of vegetables and plants. According to their structures, carotenoids are divided into two main groups (carotenes and xanthophylls). Carotenes are composed of carbon and hydrogen; for example, α-carotene, β-carotene, and lycopene. Xanthophylls are composed of carbon, hydrogen, and oxygen; for example, lutein, zeaxanthin, β-cryptoxanthin, antheraxanthin, and violaxanthin (Figure 1) [18,19]. Carotenoids have received more attention for decades due to their health-promoting roles. More specifically, some carotenoids (α-carotene, β-carotene, etc.) are the precursors of Vitamin A, which is essential for humans. Lutein and zeaxanthin are beneficial for the prevention of eye disorders [4,20]. However, carotenoids should be removed during the refining process to avoid their negative effects on the appearance of the oil.

In addition to carotenoids, high levels of chlorophyll were also found in some crude vegetable oils [21,22]. Chlorophylls are mainly composed of one cyclic tetrapyrrole, which is chelated with a centrally located Mg^2+^, and chlorophylls are responsible for the green color of vegetable oils. The main forms of chlorophyll in vegetable oils are chlorophyll a and chlorophyll b (Figure 1). Chlorophyll a is composed of a methyl group at position 7, while chlorophyll b is composed of a formyl group. These different structures result in the blue-green color of chlorophyll a and the yellow-green color of chlorophyll b [4]. During oil processing and storage, the central Mg^2+^ of chlorophyll a and chlorophyll b is easily replaced by hydrogen ions, which results in the formation of pheophytin a and pheophytin b, respectively (Figure 1) [18]. However, chlorophylls and pheophytins are prooxidants because they act as sensitizers that promote the formation of singlet oxygen under light [23,24]. Therefore, it is important to reduce the residual chlorophyll content to improve the oxidative stability of refined vegetable oils.

### 2.2. Processing Pigments

Processing pigments were typically formed by the following reactions:

(I) The oxidative polymerization of acylglycerols (monoacylglycerols, diacylglycerols, and triacylglycerols (TAG)). The major portion of vegetable oil is TAG, which is comprised of one glycerol and three groups of fatty acids. Due to the presence of unsaturated double bonds in these fatty acids, TAGs are prone to oxidative polymerization at high temperatures, which results in the formation of polar components including the oxidized TAG monomers, TAG dimers, and TAG oligomers [3]. Most of the above polar components contain carbonyl groups, conjugated double bonds, and other structures. Therefore, the oil turns brown and dark with the formation of these polar components [25]. Furthermore, Rekha et al. [26] reported that the dark compounds formed by the oxidative polymerization of monoacylglycerols/diacylglycerols were responsible for the color fixation of rice bran oil during the refining process.

(II) The non-enzymatic browning or Maillard reaction. In previous studies, non-enzymatic browning products of phospholipids (pyrrolized phospholipids) were found to rapidly enhance the color intensity of oils [27,28,29]. The Maillard reaction during the heat treatment of the oilseeds is responsible for the unique flavor of unrefined sesame and peanut oils from Asia. However, in the final stage of the Maillard reaction, the brown polymers (also named melanoidins) are formed by the condensation of the carbonyls and amines, which rapidly deepen the color of oils [30]. Furthermore, Hayashi et al. [31] reported a pseudo-Maillard rearrangement reaction of phosphatidylethanolamine (PE) and sugars, and the color of oils was deepened by the reaction.

(III) The oxidation of non-TAG. The color of oils was changed with the oxidation of non-TAG components, such as tocopherols and phospholipids [32].

(IV) The other reactions. Sono et al. [33] found that the color of oils was affected by the polyene substances generated by the degradation of phospholipids at 100 °C. In addition, the color stability of oils was also influenced by metal ions, which were discussed in the following section.

## 3. The Mechanism of Color Reversion

### 3.1. The Mechanism

**Figure 2:**
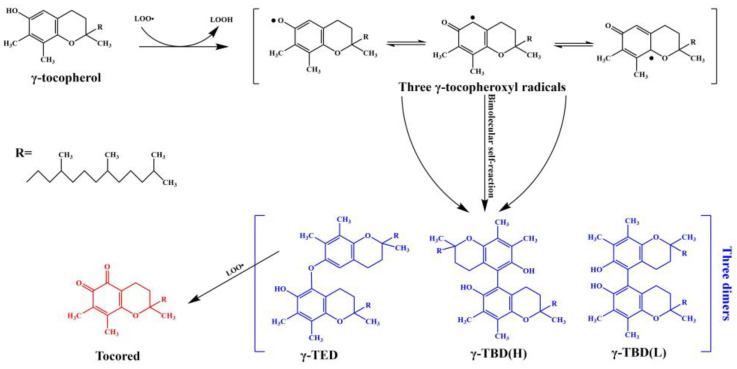
The formation mechanism of tocored. LOO•, lipid peroxide radicals; LOOH, Lipid hydroperoxides; γ-TED, 5-(γ-Tocopheroxy)-γ-tocopherol; γ-TBD(H), 5-(γ-Tocopheryl)-γ-tocopherol (H); γ-TBD(L), 5-(γ-Tocopheryl)-γ-tocopherol (L); Tocored, γ-tocopherol-5,6-quinone.

The most important natural antioxidants in oils are tocopherols, which mainly consist of α, β, γ, and δ forms [34]. γ-tocopherol is the major form of Vitamin E in many oils, such as soybean, corn germ, rapeseed, flaxseed, and sesame oils [35,36]. During the autoxidation of oils, one hydrogen atom of γ-tocopherol was transferred to the lipid peroxide radical, followed by the formation of three γ-Tocopheroxyl radicals, which were stabilized by resonance. Subsequently, these tocopherol radicals were reacted with each other (bimolecular self-reaction) to form three dimers, namely 5-(γ-Tocopheroxy)-γ-tocopherol (γ-TED), 5-(γ-Tocopheryl)-γ-tocopherol (H) (γ-TBD(H)), and 5-(γ-Tocopheryl)-γ-tocopherol (L) (γ-TBD(L)) [37,38,39]. Ultimately, γ-TED was reacted with peroxide radicals to form tocored [40]. The physicochemical properties of these compounds were summarized in Table 1, and the formation mechanism of tocored was shown in Figure 2. Tocored is a kind of *ortho*-quinones, and it tends to be yellow at low concentrations and red at high concentrations [35]. Interestingly, the vegetable oil is endowed with an orange-red color due to the presence of tocored, which is consistent with the characteristic of color-reverted oils [5].

The relationship between tocored and color reversion was investigated by Komoda et al. [12,13]. Firstly, molecular distillation was adopted to remove most unsaponifiable matters from soybean oil, and tocored was found in the unsaponifiable matters of the first distillation fraction. Interestingly, the color reversion of the oil stripped of most unsaponifiable matters was significantly restrained compared with the untreated soybean oil. Subsequently, the soybean oil was purified by molecular distillation and activated carbon column filtration to remove most impurities (such as unsaponifiable matters, tocopherols, and pigments). α-tocopherol, α-tocopherylquinone, γ-tocopherol, and tocored were then added back into the purified soybean oil in different combinations, followed by the accelerated color reversion tests for these purified oils at 100 °C. The results showed that the purified oils containing tocored showed a significant color reversion. In other words, the color intensity of the purified oils increased with the increase in tocored content. Therefore, tocored was identified as a color-reverted substance. In addition, the authors also found that, when the moisture content of the raw soybean seeds was increased from 12% to 18%, the content of tocopherols in degummed oil was reduced from 1430 mg kg^−1^ to 720 mg kg^−1^. However, the content of tocored in degummed oil was increased from 12.5 mg kg^−1^ to 83.7 mg kg^−1^, which enhanced the color intensity of the degummed oil and the probability of the color reversion of refined oil. This was consistent with the findings of Tomita et al. [11].

**Figure 3:**
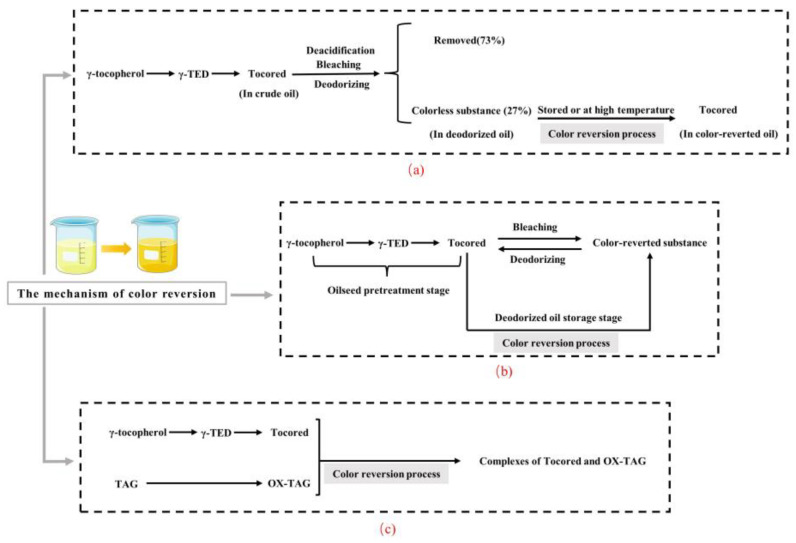
The proposed mechanisms of color reversion in refined vegetable oils. (**a**), the mechanism proposed by Komoda et al. [13], and tocored was regarded as a color-reverted substance. (**b**), the mechanism proposed by Lai et al. [14], and tocored was regarded as a precursor substance of color reversion. (**c**), the mechanism proposed by Dr. Zhang [16], and the complexes of tocored and OX-TAG were regarded as the color-reverted substances. γ-TED, 5-(γ-Tocopheroxy)-γ-tocopherol; Tocored, γ-tocopherol-5,6-quinone; TAG, triacylglycerols; OX-TAG, oxidized triacylglycerols.

In order to investigate the influence mechanism of tocored on the color reversion of soybean oil, the contents of tocored in crude, neutralized, bleached, deodorized, and color-reverted oils were determined, respectively. The results indicated that the contents of tocored in crude, neutralized, bleached, and deodorized oils were 169.2 mg kg^−1^ (100%), 99.8 mg kg^−1^ (59%), 2.0 mg kg^−1^ (1%), and 0 mg kg^−1^ (0%), respectively. However, the content of tocored in color-reverted oil was 46.5 mg kg^−1^ (27%). These results also showed that, during the oil refining processes (deacidification, bleaching, and deodorization), 73% of tocored in the crude oil was removed, and 27% of tocored was transformed into a colorless substance as well as remaining in the deodorized oil. On the contrary, when the deodorized oil was stored at room temperature or high temperatures, the colorless substance was retransformed into tocored, and the color of the oil was deepened. The above mechanism was illustrated in Figure 3a, and tocored was regarded as a color-reverted substance in the mechanism. The transformation of the tocored into the colorless substance might be explained by the fact that a quinone methide-like substance was formed during the heat treatment of tocored in unsaturated lipids, and the quinone methide was then combined with the lipid radical to form an adduct without color [41].

The contents of tocored in degummed, neutralized, bleached, deodorized, and color-reverted soybean oils were also analyzed by Lai et al. [14], and a different mechanism of color reversion was proposed. The authors found that although the deodorized oil contained more tocored than that of the color-reverted oil, its color was lighter than that of the color-reverted oil. Therefore, tocored was considered a precursor substance of color reversion instead of a color-reverted substance. This conclusion was significantly different from that of Komoda et al. [13]. Moreover, the tocored content of the degummed oil was gradually reduced after the deacidification and bleaching processes. Since the color of the bleached oil was closely related to the highest red color value of the color-reverted oil, it was speculated that tocored was converted into the color-reverted substance by the oxidation of the active clay during the bleaching process. In contrast, during the deodorization process, the tocored content of the bleached oil gradually increased, and the color of the oil became light. According to the thermodynamic analysis, the high temperature during the deodorization process was beneficial for the conversion of the color-reverted substances into tocored, and the above reaction was inverted during the subsequent storage of the deodorized oil at room temperature. The above mechanism was illustrated in Figure 3b, and tocored was regarded as a precursor substance of color reversion in the mechanism.

A different viewpoint on the qualitative study of the color-reverted substance was also proposed by Dr. Zhang [16]. Firstly, α-tocopherol and γ-tocopherol were added to the purified corn oil (mainly triacylglycerols), respectively, followed by accelerated oxidation. These results indicated that color reversion was only present in the purified oil containing γ-tocopherol, which also suggested that color reversion is related to γ-tocopherol. Subsequently, the author found that the colored substances obtained after the separation, purification, and identification of color-reverted corn oil were mainly tocored and OX-TAG, which accounted for 3.18% and 39.6% of the colored substances, respectively. Therefore, the author suggested that the color reversion of the oil was attributed to the combination of tocored and OX-TAG. During the autoxidation of the oil, a large number of free radicals and OX-TAG were formed. Meanwhile, γ-tocopherol was oxidized to tocored by trapping free radicals. Subsequently, the intermolecular hydrogen bonds were formed by the interaction of the carbonyl group in tocored with the hydroxyl group in OX-TAG, which resulted in the formation of a large conjugated system. In addition, the chromogenic ability of the chromophore in the system was increased, and the absorption wavelength of the system was shifted to long wavelengths, which increased the absorption intensity. Ultimately, the color intensity of the system was enhanced, which was known as the color reversion phenomenon. The above mechanism was illustrated in Figure 3c, and the complexes of tocored and OX-TAG were regarded as the color-reverted substances in the mechanism.

Although the above mechanisms suggested that the formation of tocored was important for the color reversion process, the role of tocored in the process was controversial (precursor, partial, or color-reverted substance). In addition, at present, knowledge about the mechanism of color reversion is also limited due to the complex transformations of tocored in oil systems [35]. Tocored has been shown to have antioxidant activity [42,43], and it might be oxidized during the color reversion process [5]. Significantly, the effect of the oxidation products of tocored on color reversion should not be ignored, as the contribution of the oxidation products of tocored to color reversion is not yet fully understood. The thermodynamic degradation of tocored in the model system (methyl linoleate) was investigated by Zheng et al. [5]. The chromatograms of the samples detected with UPLC-QTOF-MS indicated that the tocored was oxidized into unknown substances. Unfortunately, the isolation and identification of the unknown substances were not solved, and the degradation pathway of tocored was also not confirmed. Overall, future work is needed to elucidate the oxidation pathways of tocored in oil systems, which could provide new insights into the analysis of the mechanism of color reversion [44].

### 3.2. The Relationship between Color Reversion and Oil Oxidation

The formation of tocored was attributed to the capture of free radicals by γ-tocopherol, and the free radicals were generated by the oxidation of the oil [23,40]. Therefore, the oxidation of the oil was considered to be the main reason for color reversion. In other words, the oxidation of the oil was a necessary but insufficient condition for color reversion. The process of color reversion was accompanied by oxidation. However, the oxidation did not necessarily result in color reversion. The prediction equations for color reversion at room temperature were proposed by Li et al. [1]. The quantitative relationship between color reversion and the oxidation of oil was demonstrated, and the theoretical support for restraining color reversion was also provided. Color reversion was an extensive and complex process, which resulted in the poor quality of the oil. The comparison of the physicochemical properties of the deodorized oil and the color-reverted oil is presented in Table 2. To solve the issue of color reversion, the analysis of the color-reverted substances and their transformation pathways is still worthy of further exploration.

## 4. Minor Components Affecting Color Reversion

Most refined vegetable oils are typically composed of at least 99% TAG [46]. Despite the fact that pure TAG is colorless and odorless, it is prone to oxidative polymerization to produce coloring substances [3]. It is worthy of being mentioned that the color change caused by the derivatives of TAG only occurs in rancid oils. By contrast, color reversion often occurs in freshly prepared oils. Therefore, the derivatives of TAG were not identified as the color-reverted substances, which was consistent with the report of Komoda et al. [12]. In addition to TAG, refined vegetable oils also contain some minor components, such as phospholipids and metal ions, which play an important role in color reversion and are discussed as follows.

### 4.1. Phospholipids

Phospholipids are defined as a class of lipids that contain phosphorus in their structure, and their presence is detrimental to the color stability of the oil [45]. In addition, color reversion is often observed in oils with high phospholipid content. The relationship between the phosphorus content and the degree of color reversion in soybean oil was investigated by Sun et al. [47]. The authors found that the higher the phosphorus content of the deodorized oil, the more pronounced the color reversion of the oil. To study the effect of phospholipids on color reversion, lecithin was added to the medium-chain TAG system, and the color of the system was deepened in the accelerated color reversion tests [16]. According to previous reports, the color change of oils caused by phospholipids was attributed to the following reactions:

(I) The non-enzymatic browning of phospholipids. During the deodorization process of vegetable oils, when the temperature was increased from 80 °C to 160 °C, PE was reacted with the decomposition products of the peroxides to form the pyrrolized phospholipids, which resulted in an increase in oil color intensity [28]. Moreover, the high polar browning color products were formed from the unsaturated phospholipids at 180 °C, which rapidly enhanced the color intensity of the oil [27,29].

(II) The degradation of phospholipids. Sono et al. [33] found that soybean lecithin dissolved in isooctane was degraded into the coloring substances after being heated at 100 °C for 10 h, and polyene substances were separated from the coloring substances by silica gel column chromatography. Therefore, the polyenes formed by the degradation of phospholipids were considered to darken the oil.

(III) The pseudo-Maillard rearrangement of PE and sugars. Hayashi et al. [31] found that when sugar and PE were dissolved in octane solution in a 1:2 molar ratio, as the temperature increased, the sugar interacted with PE to form the pyridinium derivatives, which resulted in the formation of a brown solution. Since the formation of the derivatives was accompanied by the cleavage and rearrangement of the carbon at the 1- and 2-positions in the sugar, the reaction was called the pseudo-Maillard rearrangement reaction.

(IV) The oxidation of phospholipids. During the autoxidation of phospholipids, the amino groups of phospholipids were condensed with the aldehydes to form dark substances, which were known as melanophosphatides and rapidly deepened the color of the oil [32].

In addition to the color change, the presence of phospholipids is also detrimental to the flavor and oxidative stability of the oil. Therefore, most vegetable oils are refined to remove almost all of the phospholipids [48]. However, color reversion still occurred in refined vegetable oils with low phospholipid content, and there was also a lack of conclusive evidence that phospholipids were color-reverted substances. Therefore, phospholipids were believed to be an influencing factor in color reversion in this review. In addition, further research was still needed to clarify the influence mechanism of phospholipids on color reversion.

### 4.2. Metal Ions

Trace metal ions in oils, such as iron (Fe^3+^, Fe^2+^) and copper (Cu^2+^), are introduced by raw oilseeds or by contamination from the metal equipment used for the processing and storage of the oil [49,50]. The color of the oil was affected by metal ions in the following three primary ways:

(I) Metal ions were reacted with lipids and nonhydratable phospholipids (NHP) to generate lipid alkyl radicals and phospholipid metal complexes, respectively. The color reversion of the oil was accelerated by these products through the promotion of oil oxidation [47].

(II) Metal ions were combined with the pigments in the oil to form chelates, and the color intensity of the oil was reduced. However, when the oil was stored at room temperature or heated, the chelates were broken down into pigments, which resulted in the color reversion of the oil [51].

(III) If the acid value of the oil was too high, Fe^3+^ was compounded with FFA to produce iron salts of fatty acids, which made the oil red in color [52].

In general, the metal ion levels in crude vegetable oils are 2–15 mg kg^−1^ and less than 1 mg kg^−1^ in refined vegetable oils [49]. However, even trace metal ions can act as prooxidants to increase the rate of oil oxidation [23,53]. Since oxidation is the main reason for color reversion, the presence of metal ions is considered to have a catalytic effect on color reversion [54]. The same conclusion was obtained with the wine, as iron and copper ions could catalyze the oxidation of phenols to quinones, which led to the change in wine color [55]. Harada et al. [56] suggested that the ability of metal ions to catalyze the color reversion of oils was decreased as follows: Cu^2+^ > Fe^3+^ > Fe^2+^. Additionally, the metal ions should be removed as much as possible during the conventional refining process (degumming, deacidification, and bleaching). Furthermore, the equipment, pipelines, instruments, and valves adopted in oil processing should be composed of stainless steel to avoid the extra introduction of metal ions. Previous studies have also shown that, when the concentrations of Fe^2+^ and Cu^2+^ in the oil were less than 0.1 mg kg^−1^ and 0.01 mg kg^−1^, respectively, color reversion could be significantly restrained [57,58]. In conclusion, to ensure the quality of refined oils, special attention should be paid to the control of metal ions at every stage, from the harvesting of oilseeds to the canning of refined oils.

## 5. Restraining Measures for Color Reversion

In general, traditional oil processing consists of the following steps:

(I) The selection of oilseeds to ensure the quality of the refined oil and reduce energy consumption during subsequent processing.

(II) The pretreatment of oilseeds to improve oil yield. In particular, some oilseeds (sesame and peanut) were roasted to obtain flavored oils in Asia.

(III) The oil extraction to obtain crude oil and cake, which is used for animal feed or protein extraction.

(IV) The refining process to obtain a high-quality refined oil with minimal undesirable components. For example, degumming to remove phospholipids, deacidification to separate FFA, bleaching to remove pigments and residues formed in the previous refining processes, and deodorization to remove volatile components.

(V) The storage of refined oil to ensure its oxidative stability during transport and consumption.

The color reversion of oils was largely determined by the processing conditions described above, as these conditions were crucial for the contents of tocopherol, phospholipids, and metal ions in oils. In particular, oil oxidation (especially the oxidation of γ-tocopherol) is the main reason for color reversion, and phospholipids and metals are considered influencing factors. Therefore, the quality control of oilseeds, the pretreatment methods, the technical parameters of the oil extraction and refining processes, as well as the storage conditions, were compiled in this review to restrain color reversion.

### 5.1. The Quality Control of Oilseeds

Quality control of oilseeds is defined as the selection of the region, cultivar, or maturity of the raw seeds, the adjustment of the moisture content, and storage conditions. The degree of color reversion in oils was significantly influenced by the quality of the oilseeds [59,60,61]. Taking the example of soybeans, which were commonly used to study the phenomenon of color reversion, the degree of color reversion of refined soybean oil composed of different regions or cultivars of soybean seeds was different. This could be explained by the fact that the environmental condition, genotype, and moisture content all affected the tocopherol content of soybean seeds [62]. In particular, when the moisture content of soybean seeds exceeded 12%, the loss of tocopherols in soybean oil increased sharply, which might result in an increase in the degree of color reversion of the oil [11,12]. Since the NHP (mainly phosphatidic acid) content of immature soybean was three times that of mature soybean, the color of the oil obtained from the immature soybean was unstable [63]. In addition, during storage and transportation, various redox enzymes in the oilseeds resulted in the oxidative decomposition of tocopherols. Recently, tocopherol oxidase was extracted from the corn germ by Zheng et al. [61], and the catalytic effect of this oxidase on γ-tocopherol was also investigated. The results showed that γ-tocopherol was oxidized by this oxidase to form tocored in the presence of lecithin. As the formation of tocored was thought to be closely related to color reversion, this finding might provide new insights into color reversion from the enzyme perspective. To restrain the color reversion of soybean oil, mature soybeans were selected as raw materials, and the moisture of the soybean seeds should be adjusted to no more than 12% at appropriate storage conditions.

### 5.2. Pretreatment Process

Before the crude oil is extracted, oilseeds such as soybeans are often subjected to a series of pretreatments, such as cleaning, drying, cracking, conditioning, and flaking. During the conditioning process, steam is often used to heat and moisten the soybeans to obtain the optimum plasticity for the production of soybean flakes. Chu and Lin [15] found that the color reversion of refined soybean oil was dramatically restrained after the steam pretreatment of soybeans. On the one hand, the enzymatic oxidation of γ-tocopherol was inhibited in the steam-treated soybeans. On the other hand, steam pretreatment resulted in a decrease in NHP and an increase in hydratable phospholipids (HP) in crude oil by inhibiting the activity of phospholipase D, which catalyzed the conversion of HP to NHP [64]. However, when the steam treatment was applied to soybean flakes, the color reversion of soybean oil was not completely restrained. The results indicated that the enzymatic reactions related to γ-tocopherol oxidation happened before or during the flaking process, and this finding was consistent with the report of Lai et al. [14]. By contrast, conventional toasting was less effective than steam pretreatment in restraining color reversion. In addition, Chu and Lin [15] also found that, when the total content of γ-tocopherol and γ-TED in crude oil exceeded 550 mg kg^−1^, the probability of color reversion of refined soybean oil was low, and this value could be used to predict the color quality of the oil. The effects of raw soybean pretreatment on the content of risky substances (γ-tocopherol, phospholipid) and the degree of color reversion in soybean oil are presented in Table 3.

### 5.3. Oil Extraction Process

The widely used technology for oil extraction from seeds is solvent extraction [65]. The liquid part obtained by solvent extraction is known as the mixed oil, which is composed of the volatile solvent, the non-volatile oil, and some undesirable compounds (phospholipids, sugars, proteins, etc.). The removal of these undesirable compounds was considered to contribute to the reduction of color reversion. In addition, more attention should be paid to the temperature at which the mixed oil was evaporated to remove the solvent. When the evaporation temperature in the leaching workshop was higher than 105 °C, the color of the oil was extremely unstable. Therefore, low temperatures or increased vacuum in the evaporation phase of the mixed oil should be adopted [66].

### 5.4. Refining Process

#### 5.4.1. Degumming

Degumming is the first step of the refining process, and most phospholipids, trace metal ions, and mucilaginous substances are removed during the degumming process [67]. In general, the phospholipids commonly found in vegetable oils are divided into HP and NHP [68]. The main HP in oils are phosphatidylcholine and phosphatidylinositol, and the main NHP are the calcium and magnesium salts of phosphatidic acid and PE [69]. Since phospholipids (especially in NHP) have been confirmed to promote the color reversion of oils, they should be removed as much as possible. HP can be removed by water degumming (washing the oil with water). For NHP, food-grade phosphoric acid or citric acid is often used to convert it into HP, followed by washing with water, which is known as acid degumming [24]. It is worthy of being mentioned that insufficient addition of acid could result in the color reversion of oils, and excessive addition of acid also resulted in an increase in the phosphorus content of the oil. In general, 0.1–0.3% of an 85% phosphoric acid solution or 0.1–1.0% of a 30% citric acid solution was used for acid degumming [49]. In addition, Dai [70] found that, when the amount of residual phosphorus in refined vegetable oils was above 5 mg kg^−1^, the color of the oil was unstable during storage. To avoid the effect of phospholipids on color reversion, the phosphorus content of degummed oil, bleached oil, and refined oil should be controlled within 12 mg kg^−1^, 2 mg kg^−1^, and 1 mg kg^−1^, respectively [71].

#### 5.4.2. Deacidification

The deacidification process refers to chemical refining using alkali and physical refining by steam distillation. In chemical refining, FFA is neutralized by an alkali (usually sodium hydroxide). Several studies have demonstrated that the effect of alkali dosage on color reversion is not significant [72]. However, insufficient addition of alkali solution could result in high acid value and low oxidative stability of the oil, as FFA acted as prooxidants by promoting the decomposition of hydroperoxides [73]. On the other hand, more alkali could attack the neutralized oil, which was called “parasitic” saponification and resulted in a decrease in neutralized oil yield [74]. Therefore, besides the theoretical amount of alkali solution required to neutralize FFA, the addition of excess alkali solution should be limited to 0.1–0.25% [49,57]. Significantly, FFA reacted with alkali to form insoluble soap, which could adsorb impurities such as phospholipids, pigments, and metal ions. Therefore, the presence of soap was considered to have a catalytic effect on color reversion. To ensure the color stability of the oil, the residual soap amount of the neutralized oil should be controlled to less than 40 mg kg^−1^ [75].

For physical refining, the neutralization process with alkali is not involved, and FFA is removed by steam distillation. Therefore, physical refining was considered to be more environmentally friendly and economical than chemical refining. However, physical refining is often carried out at a higher temperature (270 °C) than chemical refining (220–250 °C), which results in higher requirements for the quality of the oil used for physical refining than that of chemical refining [32,76]. To avoid the color reversion or color fixation caused by the phospholipids in physically refined oils, the phosphorus content of the oil used for physical refining should be less than 15 mg kg^−1^, and preferably less than 5 mg kg^−1^ [77].

#### 5.4.3. Bleaching

Unwanted substances such as chlorophylls, carotenoids, trace amounts of soap, metal ions, and some contaminants (pesticides, etc.) are often adsorbed by the adsorbents in the bleaching process. The adsorbents commonly used in the oil industry are natural bleaching earth, acid-activated earth, attapulgite clay, or a combination of these substances [78].

Natural bleaching earth is composed of silicates such as bentonite and sepiolite [78,79]. Acid-activated earth was obtained from calcium bentonite, which was activated by hydrochloric or sulfuric acid, followed by washing and drying to a moisture content of 10–15% [49]. The purpose of activation was to increase the specific surface area of the earth to 250–350 m^2^/g and the bleaching efficiency. In the traditional theory of bleaching, the higher the activity of acid-activated earth, the stronger its ability to adsorb organic impurities. However, it has been shown that acid-activated earth with high activity has a negative effect on the quality of the oil; for example, their presence is catalytic for the formation of 3-monochloropropane-1,2-diol esters and the color reversion of oils [71,80]. Therefore, the activity of acid-activated earth should be around 50 mmol/kg to eliminate these negative effects [71]. 

Even though the decolorization rate of attapulgite clay was not as good as that of acid-activated earth, it could keep the acid value and color of bleached oil stable. Zhang et al. [51] found that the decolorization rate of soybean oil was 71.83% under the conditions of 32 min bleaching time, 110 °C bleaching temperature, 250 r/min stirring rates, and 3% attapulgite clay load. Furthermore, the degree of color reversion of the above-bleached oil was lower than that of the control oil bleached with acid-activated earth. This might be attributed to the fact that attapulgite clay contained fewer metal ions (iron and copper ions) than acid-activated earth. In conclusion, to ensure the color stability of the oil, the appropriate adsorbent should be used according to the quality of the neutralized oil and the characteristics of different adsorbents.

#### 5.4.4. Deodorization

Deodorization is a steam distillation at high temperatures (220–250 °C) and vacuum pressure (0.3–0.6 kPa). The objective of deodorization is to remove the substances that affect the sensory and quality of the oil, for example, FFA (in physical refining), off-flavor components, oxidation products, and other contaminants (polycyclic aromatic hydrocarbons, pesticides, etc.). In addition, some bioactive components, such as tocopherols and phytosterols, are also unavoidably degraded during deodorization, which increases the probability of color reversion [74]. Therefore, the methods to prevent color reversion in three periods (before, during, and after deodorization) are discussed in this section.

Before deodorization, oxygen, phospholipids, soaps, and metal ions that could accelerate oil oxidation and color reversion should be removed as much as possible. For example, the oil was heated at 85–90 °C under a vacuum or passed through a deaerator to avoid the negative effects of oxygen [81]. A 0.03% citric acid or phosphoric acid solution was also added to the temporary storage tank, which was beneficial in retarding color reversion by chelating the metal ions in oils [54,57]. During the deodorization, the reaction temperature and time should be reasonably matched. Taking tocopherol as an example, the retention of tocopherol decreased with the increase in deodorization temperature (210–270 °C) and deodorization time (60–120 min). In particular, the degradation of γ-tocopherol was significant, which was considered to be associated with color reversion [6,13,14,82]. Thus, a long deodorization time at low temperatures or a short deodorization time at high temperatures was often adopted [17]. Decap et al. [83] suggested that the steam could be replaced by using nitrogen as the stripping gas, which could avoid the oxidation of the active components and improve the oxidation and color stability of the oil. After deodorization, the oil is usually cooled to 60–80 °C [49]. However, Li [84] reported that the outlet temperature of deodorized oil should be kept below 45 °C; otherwise, the oil at high temperatures is exposed to the metal storage tank and oxygen, which could accelerate the oxidation and color reversion of the oil.

#### 5.4.5. Dewaxing

Waxes are high-melting esters of fatty alcohol and fatty acids with low solubility in oils [85]. Although the oils containing waxes were not detrimental to health, the presence of waxes affected the appearance of the oil, especially in winter when the precipitation of dissolved waxes could give the oil a hazy appearance [74]. Therefore, the oil needed to be dewaxed to meet the demands of consumers. The dewaxing process was usually carried out at 0–19 °C for at least 6 h, followed by filtration with a filter aid to remove the crystallized, high-melting components [32,49]. During the process, no significant chemical reaction happened, and the color of the oil was not significantly affected. At the same time, no report on the relationship between color reversion and wax (or dewaxing) has been found to date.

#### 5.4.6. Moderate Refining

To avoid the color reversion of oils, some bioactive components with antioxidant activity should be retained in oils as much as possible, and the use of substances that could trigger the oxidation of oils should be minimized during the refining process. This was consistent with Wang’s proposal of moderate refining in the Chinese edible oil industry, which ensured the maximum reservation of nutrients and the minimum formation of deleterious components [86]. The color change of commercially refined vegetable oils and unrefined oils (without any refining treatment or only lightly degummed) during six months of storage at room temperature was investigated by Dr. Zhang [16]. The author found that color reversion was rarely observed in unrefined oils. By contrast, color reversion was present in almost half of the refined oils. These findings also indicated that moderate refining was an effective method to retard color reversion.

In terms of moderate refining technology for vegetable oil, each oilseed needs to formulate a corresponding processing technology according to its characteristics. The personalized technical solution is the development trend in the edible oil industry. However, at present, the moderate refining technology of various oilseeds (rice bran, cottonseeds, Camellia oleifera seeds, etc.) remains to be further improved. The related equipment and the efficient utilization of by-products also need to be further studied [87].

### 5.5. Storage of Refined Vegetable Oils

#### 5.5.1. External Environment for Oil Storage

The rate of color reversion in oils was greatly affected by temperature, light, and air [88]. In the range of 20 °C to 60 °C, when the storage temperature of edible oils was increased by 15 °C, the speeds of oxidation and color reversion were increased by two and four times, respectively. Koo [89] found that color reversion could be effectively retarded by reducing the oil temperature in the storage tank to below 50 °C in the summer. However, Patterson [59] found that color reversion in oils was common after six months of storage at around 40 °C. The effect of light on the color reversion of refined corn oil was investigated by Sun et al. [58]. The oils were sealed and stored at room temperature, followed by exposure to varying degrees of light. The authors found that the rate of color reversion of oils increased with the increase in light intensity, which also indicated that the color reversion was catalyzed by light. Therefore, the oil was recommended to be stored away from light. One of the effective measures to restrain oil oxidation and color reversion was to minimize the contact between oil and air [66,90]. Therefore, the liquid level of the oil storage tank or bottle should be maintained as high as possible and flushed with nitrogen [32,49]. However, the protective effect of the nitrogen was limited after the oil storage bottle was opened. As a result, nitrogen was usually used in combination with other methods to maintain the quality of the oil.

#### 5.5.2. The Use of Antioxidants

The color reversion of refined vegetable oils is accompanied by oxidation [1,13,14]. Therefore, the use of antioxidants seems to be another effective way to solve this issue. Antioxidants are divided into synthetic antioxidants and natural antioxidants according to their sources. Tert-butylhydroquinone (TBHQ), butylated hydroxyanisole, and butylated hydroxytoluene are synthetic antioxidants widely used in edible oils. Among these, TBHQ can provide a hydrogen atom to a radical formed during the oxidation process to interrupt the free radical chain propagation process [91]. The oxidized TBHQ, after donating one hydrogen atom, is turned into a free radical intermediate, which is stabilized by the formation of resonance delocalization of electrons within the aromatic ring [92]. Although TBHQ is highly efficient and stable, the allowable addition of TBHQ is strictly limited in most countries. For example, the maximum allowable amount of TBHQ in edible oils is 200 mg kg^−1^ in the United States, Australia, and the European Union and is not allowed in Japan [93]. Shen [72] found that the degree of color reversion of oils decreased with the addition of TBHQ from 0 mg kg^−1^ to 60 mg kg^−1^. However, TBHQ with more than 60 mg kg^−1^ showed minor effects on the inhibition of color reversion. More crucially, the safety of these synthetic antioxidants has been questioned because of their possible toxic effects [93,94]. Therefore, more attention has been focused on the substitution of synthetic antioxidants with natural antioxidants [24,95].

**Figure 4:**
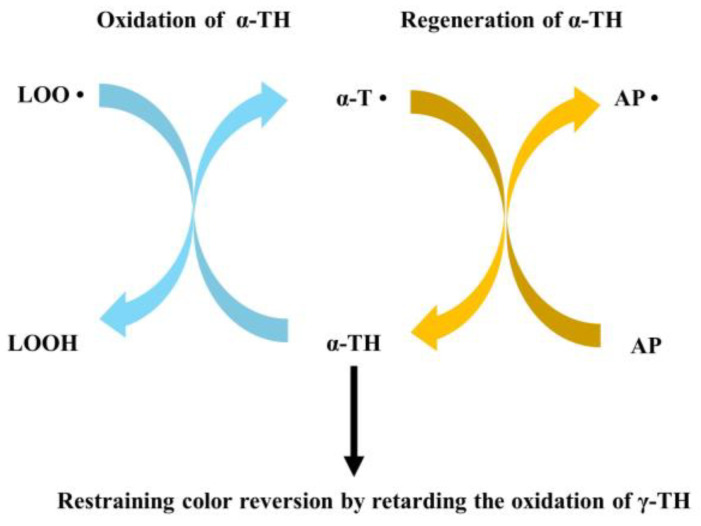
The possible restraining pathway of ascorbyl palmitate on color reversion. LOO•, lipid peroxide radicals; LOOH, Lipid hydroperoxides; α-TH, α-tocopherol; α-T•, α-tocopherol radicals; AP, ascorbyl palmitate; AP•, ascorbyl palmitate radicals.

Ascorbyl palmitate (AP), a natural antioxidant [95], was a class of oil-soluble palmitate ester of ascorbic acid and acted as an oxygen scavenger and synergist. You et al. [96] reported that the degree of color reversion of refined corn oil decreased with increasing concentrations of AP in the range of 0 mg kg^−1^ to 200 mg kg^−1^. This might be due to the fact that, during the oxidation of oils, α-tocopherol was earlier oxidized than γ-tocopherol. The oxidized α-tocopherol is then restored by AP, thus slowing down the oxidation of γ-tocopherol and restraining the color reversion of oils (Figure 4) [93]. The storage stability of refined corn oil with the addition of AP and TBHQ was compared by Wu et al. [90]. The authors found that, when AP and TBHQ were added at 150 mg kg^−1^ and 50 mg kg^−1^, respectively, the effects on the acid value, peroxide value, and degree of color reversion of the samples were almost the same. However, AP had a slightly higher effect on the maintenance of tocopherol content in the oil than TBHQ.

Natural antioxidants are good alternatives to synthetic antioxidants because consumers are concerned about the safety of synthetic compounds. However, natural antioxidants have some limitations and disadvantages, such as low thermal stability and expensive costs. Therefore, in order to achieve the commercial sustainability of natural antioxidants, more efficient and economical methods for the extraction of natural antioxidants from plant matrices should be developed [93,97].

## 6. Conclusions and Prospects

The market for refined vegetable oils has been plagued by the phenomenon of color reversion. A comprehensive understanding of the mechanism of color reversion and the effective restraining of this phenomenon is of increasing interest. As discussed in many studies, the color reversion of oils is involved in a large and complex system. Although some studies have shown that γ-tocopherol oxidation is the main reason for color reversion, the proposed mechanisms of color reversion are controversial. Consequently, the molecular action of the oxidized derivatives of γ-tocopherol (especially tocored) in lipid systems is worth exploring, as this could provide new insights into the mechanism of color reversion. Moreover, further research should also focus on elucidating the mechanism of the effect of phospholipids and metal ions on color reversion. Despite the achievements, the issue has not been satisfactorily solved. An efficient and economical method to restrain color reversion is still needed to be developed.

## Figures and Tables

**Table 1:** Summary of some physicochemical properties of γ-tocopherol and its oxidized derivatives.

Compound	Abbreviation	Molecular Formula	Molecular Weight (g/mol)	IUPAC Name	PubChem CID	UVmax in Methanol (nm)
γ-Tocopherol	–	C_28_H_48_O_2_	416.7	(2R)-2,7,8-trimethyl-2-[(4R,8R)-4,8,12-trimethyltridecyl]-3,4-dihydrochromen-6-ol	92729	211, 257, 298
5-(γ-Tocopheroxy)-γ-tocopherol	γ-TED	C_56_H_94_O_4_	831.3	2,7,8-trimethyl-2-(4,8,12-trimethyltridecyl)-5-[[2,7,8-trimethyl-2-(4,8,12-trimethyltridecyl)-3,4-dihydrochromen-6-yl]oxy]-3,4-dihydrochromen-6-ol	11735491	203, 293
5-(γ-Tocopheryl)-γ-tocopherol (H)	γ-TBD(H)	C_56_H_94_O_4_	831.3	cis-5-[6-hydroxy-2,7,8-trimethyl-2-(4,8,12-trimethyltridecyl)-3,4-dihydrochromen-5-yl]-2,7,8-trimethyl-2-(4,8,12-trimethyltridecyl)-3,4-dihydrochromen-6-ol	13517848	203, 302
5-(γ-Tocopheryl)-γ-tocopherol (L)	γ-TBD(L)	C_56_H_94_O_4_	831.3	trans-5-[6-hydroxy-2,7,8-trimethyl-2-(4,8,12-trimethyltridecyl)-3,4-dihydrochromen-5-yl]-2,7,8-trimethyl-2-(4,8,12-trimethyltridecyl)-3,4-dihydrochromen-6-ol	–	205, 293
γ-Tocopherol-5,6-quinone	Tocored	C_28_H_46_O_3_	430.7	(2R)-2,7,8-trimethyl-2-(4,8,12-trimethyltridecyl)-3,4-dihydrochromene-5,6-dione	101701364	208, 284, 471

Adapted from Zheng et al. [35].

**Table 2:** Comparison of physicochemical properties of deodorized oil and color-reverted oil.

Physicochemical Properties	Deodorized Oil	Color-Reverted Oil
Color (red value)	1.3	4.2
Color (yellow value)	8	18
Peroxide value (meq kg^−1^)	0.42	0.6
p-Anisidine value	2.98	3.28
Total oxidative value	0.789	1.259
Unsaponifiable matter (%)	0.45	0.40
Saponification value (mg KOH g^−1^)	194.62	194.28
Total polyphenols (mg kg^−1^)	1.85	1.64

Adapted from Atta and Al-Okaby [45].

**Table 3:** The effects of raw soybean pretreatment on the content of risky substances (γ-tocopherol, phospholipid) and the degree of color reversion in soybean oil.

Pretreatment	Condition	Phosphorus Content (mg kg^−1^)			γ-Tocopherol (mg kg^−1^)	Oil Color (Lovibond Red/Yellow Value)		
		Crude Oil	Degummed Oil	Reduction (%)		Deodorized Oil	Color-Reverted Oil	ΔR
Control	Without any treatment	494	426	13.80	92	3.6/42	12/70	8.4
Steam	100 °C, 1 min	708	40	94.4	733	0.5/3.0	1.4/10	0.9
Steam	100 °C, 1.5 min	717	18	97.5	651	0.5/2.6	1.3/9.4	0.8
Steam	100 °C, 2.0 min	563	34	94.0	686	0.5/3.1	1.4/10	0.9
Toast	110 °C, 30 min	535	187	65.0	227	2.2/13	4.2/35	2
Toast	130 °C, 30 min	564	193	65.8	377	1.4/6.7	2.5/20	1.1
Toast	150 °C, 30 min	587	208	64.5	452	1.2/7.6	2.1/14	0.9

Reduction, % = (Pc − Pd)/Pc; Pc, Phosphorus content of crude oil; Pd, Phosphorus content of degummed oil. ΔR = Rd − Rc; Rd, Red value of deodorized oil; Rc, Red value of color-reverted oil. Adapted from Chu and Lin [15].

## Data Availability

Not applicable.
